# Familial Cases of Legg–Calvé–Perthes Disease—Hemostatic and Molecular Markers

**DOI:** 10.3390/ijms27052195

**Published:** 2026-02-26

**Authors:** Edgar Hernández-Zamora, Armando Odiseo Rodríguez-Olivas, Marlene Alejandra Galicia-Alvarado, Leonora Casas-Ávila, Erika Rosales-Cruz, Cesar Zavala-Hernández, Elba Reyes-Maldonado

**Affiliations:** 1 Genomic Medicine, Instituto Nacional de Rehabilitación “Luis Guillermo Ibarra”, Calzada México-Xochimilco 289, Arenal de Guadalupe, Tlalpan 14389, Mexico City, Mexico; lcasasa@gmail.com; 2 Instituto de Ciencias Biomédicas, Universidad Autónoma de Ciudad Juárez, Campus Cuauhtémoc, Km. 3.5 Carretera Anáhuac s/n, Anáhuac, Municipio de Cuauhtémoc 31600, Chihuahua, Mexico; orox3@hotmail.com; 3 Neurociencias Clínicas, Instituto Nacional de Rehabilitación “Luis Guillermo Ibarra”, Calzada México-Xochimilco 289, Arenal de Guadalupe, Tlalpan 14389, Mexico City, Mexico; marlenegalicia@gmail.com; 4 Morphology Department, Escuela Nacional de Ciencias Biológicas, Instituto Politécnico Nacional, Prolongación de Carpio y Plan de Ayala s/n, Col. Santo Tomás, Gustavo A. Madero 11340, Mexico City, Mexico; erika_encb@hotmail.com; 5 Clinical Pathology Laboratory, Instituto Nacional de Rehabilitación “Luis Guillermo Ibarra”, Calzada México-Xochimilco 289, Arenal de Guadalupe, Tlalpan 14389, Mexico City, Mexico; cezaher@yahoo.com.mx

**Keywords:** LCPD, etiology, genetics, environmental, metabolic

## Abstract

Legg–Calvé–Perthes disease (LCPD) is a rare disease caused by avascular necrosis of the femoral head. Although its etiology is still not fully understood, evidence suggests that heritable prothrombotic and inflammatory factors, as well as environmental factors, may be implicated in its onset and progress. The objective of this study is to describe the genetic, biochemical, and environmental factors that may be associated with the etiology of LCPD. This study was conducted in three families and included seven related patients with an LCPD diagnosis. We evaluated the following gene alterations using real-time PCR: *MTHFR*, *CBS*, *COL1A1*, *COL2A1*, *PT*, *FVL*, *FVIII*, *FIX*, *PAI-1*, *eNOS*, *IL-23R*, *TNF-α*, *RANNK*, *RANNK-L*, *OPG* and *IL-6*. Additionally, we assessed fourteen thrombophilia-associated biochemical markers, as well as environmental factors that may be associated with the etiology of LCPD in family cases. The results show different hemostatic alterations in every individual analyzed, presenting out-of-range values in one or more parameters. Concentrations of hemoglobin and fibrinogen and the FIX activity percentage showed statistically significant differences (*p* < 0.001) when compared with healthy controls. All patients presented at least one mutated allele for the *MTFHR* (rs1801133), IL-23R (rs1569922) and *OPG* (rs2073618) polymorphisms, as well as isolated cases with other genetic variants. Our results show environmental elements from every family, and hemostatic and inflammatory disorders, may be involved in the development of LCPD. Furthermore, genetic variants could contribute to the onset of the disease. This study highlights the multifactorial nature of this pathology, involving various environmental, genetic, inflammatory, and prothrombotic factors in three families that included seven patients diagnosed with LCPD.

## 1. Introduction

Legg–Calvé–Perthes disease (LCPD) is classed as a rare disease due to its low incidence and unknown etiology. LCPD presents as uni- or bilateral avascular necrosis of the femoral head (FH), which affects the range of motion of the hip to varying degrees and causes pain in the affected limb that intensifies during and after physical activity. It has a very variable incidence, ranging between 0.4/100,000 and 29.0/100,000, and it mainly occurs in males. Unfortunately, there are no data on its prevalence in Latin America, but it is considered a low-incidence disease. There are multiple theories about the etiology of LCPD; however, many remain controversial due to the lack of a foundation or reproducibility. Nonetheless, the interruption of blood flow to the FH and subsequent ischemic necrosis seem to be critical events in the development of LCPD since the pathological and structural changes characteristic of LCPD are perceptible afterward. At the moment, there is considerable evidence of the absence of blood flow to the affected FH; histological studies have also shown changes consistent with ischemic necrosis of the deep portion of the articular cartilage [1,2]. Research shows that at least two ischemic episodes might be necessary for LCPD to develop. However, studies in animal models have found that a single ischemic event produces changes similar to those found in LCPD [3,4]. Necrosis leads to the decay of the mechanical and support-related properties of the bone and articular cartilage, resulting in the deformation of the FH due to mechanical force [3,5].

There is evidence that genetic mechanisms may be involved in the etiology of LCPD, such as inheritance patterns, ranging from autosomal recessive to polygenic. While there appears to be an autosomal dominant mode of inheritance [6,7] in families with a high rate of affected individuals, Gray et al. found that the rate of occurrence of LCPD in first-, second-, and third-degree relatives combined is 1:39. They also found that the rate of occurrence is 1:26 among siblings, i.e., 35- and 50-times greater than in the general population [8]. Because of this, some authors consider the possibility that there may be a more significant association of LCPD among relatives.

Furthermore, hemostatic alterations, such as hypofibrinolysis and hypercoagulable states, have been proposed as triggering factors of LCPD. Studies present high levels of fibrinogen and factor VIII (FVIII) as possible causal factors, in addition to polymorphisms such as the factor V Leiden mutation (*FVL*) and the prothrombin 20210 (G/A) polymorphism (*PT G20210A*) as possible causal factors. C667T polymorphisms of methylenetetrahydrofolate reductase (*MTHFR C667T*) and the T833C polymorphism of cystathionine beta-synthase (*CBS T833C*), which are characterized by increased levels of homocysteine (Hcy) in the blood, are proposed as causative agents of LCPD. Although no apparent relationship between these polymorphisms and LCPD was found, it has been reported that *MTHFR* polymorphisms are implicated in a wide variety of thromboembolic diseases, and elevated Hcy levels have been related to osteonecrosis [9,10,11,12].

Plasminogen activator inhibitor-1 (PAI-1) is the main inhibitor of tissue plasminogen activator (t-PA) and urokinase (uPA), which are the activators of plasminogen and, therefore, of fibrinolysis PAI-1. This inhibitor is mainly produced in the endothelium, although it is also secreted by other cell types, such as adipose tissue. In addition, PAI-1 is involved in angiogenesis, and some polymorphisms in the PAI-1 gene have been related to osteonecrosis femoral head (ONFH) [13,14].

Nitric oxide (NO) participates in multiple physiological processes, such as angiogenesis, thrombosis, coagulation, and fibrinolysis. Nitric oxide synthetase is the main enzyme in NO metabolism. It has three isoforms: endothelial (eNOS), inducible (iNOS), and neuronal (nNOS). There is evidence of an association between eNOS polymorphisms and cardiovascular diseases (coronary artery disease, chronic heart failure, hypertension, atherosclerosis, stroke, renal diseases, and avascular necrosis of the FH). It has been proposed that LCPD could involve alterations in the vascularization of the FH. Therefore, sequence variations in the *eNOS* gene could alter nitric oxide synthesis and affect the progression of LCPD [15,16].

Interleukin 23 (IL-23) is a proinflammatory cytokine characterized by the binding of the IL-12 receptor (IL-12R) with the IL-23 receptor (IL-23R). However, IL-23 promotes inflammation primarily through recognition by the IL-23R. It has recently been reported that IL-23-deficient mice were resistant to collagen-induced arthritis. In addition, IL-23R has been related to different inflammatory disorders, and some of its variations have been associated with ONFH [17,18].

The bone remodeling pathway formed by RANK-RANKL-OPG is the principal mechanism controlling the equilibrium of bone formation and bone resorption. Other molecules involved in the regulation of bone metabolism are the interleukin 6 (*IL-6*) polymorphisms in these genes, which have been assessed for their participation in LCPD [12,19].

Tumor necrosis factor α (TNF-α) is also a proinflammatory cytokine with a central role in immune response, among other functions. It is related to bone remodeling since it stimulates osteoclastogenesis and simultaneously inhibits some functions of osteoblasts. Some variations in the TNF-α gene have been associated with ONFH, although no relation was found between these and LCPD [1,12,19].

Finally, other studies have described polymorphisms in the collagen type I and II genes (*COL1A1* and *COL2A1*) associated with families that include individuals with LCPD [1].

Family members not only share genes but also habits and environments. Consequently, studies that include familial cases are essential for determining whether a true genetic risk exists. Family-based research helps to clarify whether a disease is inherited (i.e., has a genetic component) or is primarily driven by shared factors such as lifestyle or diet. With respect to LCPD, very few studies have examined familial cases; therefore, this study aims to characterize the environmental factors, genetic polymorphisms, and biochemical hemostatic markers that may contribute to the etiology of LCPD in three Mexican families with multiple affected members.

## 2. Results

### 2.1. Biochemical Hemostatic Markers

Seven patients and fourteen controls were studied. Table 1 presents the results of the examination of biochemical hemostatic markers in patients and controls, including coagulation factor levels, antithrombotic proteins, fibrinogen, and homocysteine. These are metabolites that have previously been associated with thrombosis. The table also includes the Proposed Reference Values for Children (RVPC) and the Reference Values of Commercial Kits (RVCK) [20]. Values outside the reference ranges for the fourteen markers in both the patients and controls are highlighted in bold. In addition, certain characteristics are shown, such as whether individuals are adults or children and whether they present unilateral or bilateral involvement. Some adults also exhibited values above the normal range (Table 1).

**Figure 1:**
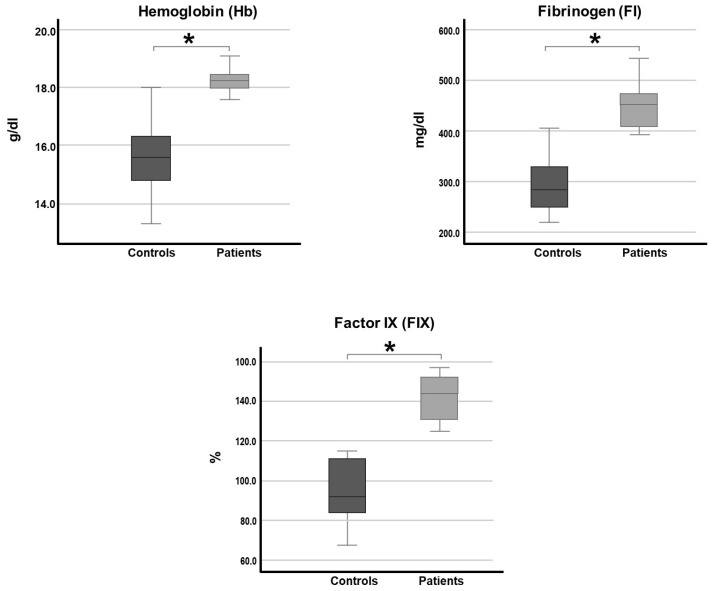
Concentration and activity of hemostatic markers in LCPD patients and controls. Medians are shown inside each box plot. Hemoglobin (Hb). Fibrinogen (FI). Factor IX (FIX). Mann–Whitney U with Rstudio 4.3, * *p* < 0.001.

Statistical analysis of the biochemical hemostatic markers was performed to determine whether significant differences existed between the groups (patients and controls). Significant differences were found in hemoglobin (Hb) levels (*p* < 0.001), fibrinogen (*p* < 0.001), and factor IX (FIX) activity percentage (*p* < 0.001) (Figure 1).

### 2.2. Molecular Markers

Table 2 summarizes the identified mutations and polymorphisms, which were selected based on their established associations with thrombosis, inflammation, and osteoarthritis in LCPD patients. In genotyping, due to the number of subjects in this study, Chi-square tests and the Hardy–Weinberg equilibrium could not be performed. Nonetheless, all participants in this study had at least one mutated allele of the MTFHR (1801133) and IL-23R (1569922) polymorphisms, and isolated cases had other genetic variants.

### 2.3. Clinical Data

**Figure 2:**
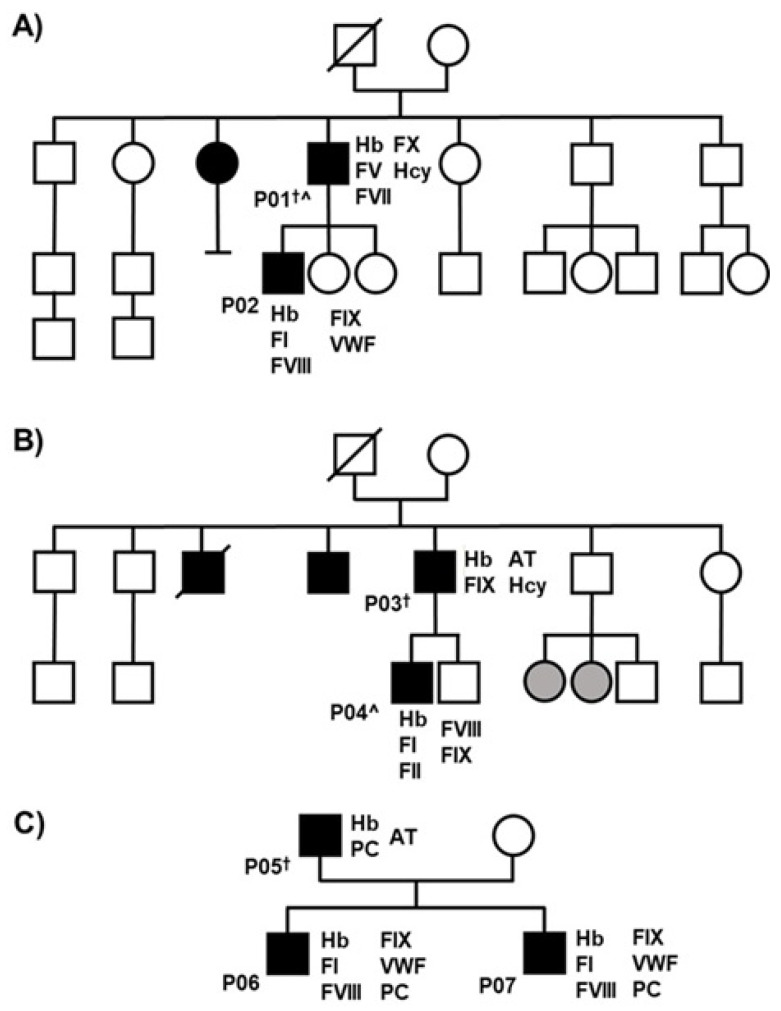
Pedigrees of the individuals with LCPD included in the study. (**A**) Family 1. (**B**) Family 2. (**C**) Family 3. Square = males. Circle = females. Slash = deceased. Black = LCPD. Grey = osteoarthritis. P = patient. Hb = hemoglobin. F = coagulation factor. VWF: Von Willebrand factor. PC = protein C. AT = Antithrombin. Hcy = homocysteine. † = adult. ^ = bilateral disease. Mentioned values are all those which were out of range.

Figure 2 shows the pedigrees of the three families included in this study. The figure highlights several relevant clinical features. Two bilateral cases were identified: P001 and P004. Flat feet were observed in three patients (P003, P004, and P005), and one patient practiced taekwondo (P001), which is a high-impact sport. In addition, Family 2 (Figure 2B) had close relatives affected by osteoarthritis.

All adult participants reported being smokers, and all patients had habitual exposure to wood and tobacco smoke. In Mexico, six socioeconomic levels have been defined, each associated with different income and consumption patterns. The population included in this study ranged between levels C and D, corresponding to the middle, lower-middle, and lower socioeconomic classes (https://www.amai.org/, accessed on 26 September 2024).

## 3. Discussion

In each of the families studied, we observed several characteristics. For example, some members suffered from osteoarthritis in Family 2, which had the highest number of patients with LCPD (Figure 2B). This pathology has been previously related to LCPD [21], so it would be of interest to examine whether there are alterations related to bone development in our population that are associated with LCPD.

LCPD is a complex disease; the lack of knowledge regarding its etiology is considered the main obstacle to its study. Different etiological factors have been indicated to be the causative agents of LCPD. Some of these were present in our population, e.g., socioeconomic deprivation, which is an important factor to consider, since a higher incidence of the disease has been observed in populations of a lower socioeconomic level. The socioeconomic level of our population ranged between middle, lower-middle, and lower class, so it can be considered to be an environmental factor and could be related to the predisposition to LCPD due to poor nutrition, urbanization, and other variables [22,23].

Another factor observed in our group of patients was exposure to wood and tobacco smoke, which several studies consider to be a relevant factor in the appearance of LCPD. According to the results of previous studies, wood or tobacco smoke may be related to alterations in hemostasis by various mechanisms [24,25,26].

Moreover, our population showed disturbances in the distribution of mechanical load, such as overdue arches or the practice of high-impact sports like taekwondo and gymnastics, which could lead to the development of LCPD due to the discrepancy of the forces applied on the hip and femur, as well as venous occlusion [27,28].

A family study was performed with the presence of related patients, so it is proposed that genetically inherited factors could cause LCPD. Although no evidence was found that relates the polymorphisms studied to LCPD, the development of this disease could involve other genetic alterations [6,7].

Regarding laboratory studies, all our patients presented high hemoglobin levels (*p* < 0.001), which are linked to increased blood viscosity and subsequent thrombotic events [29,30,31]. In addition, our results show different hemostatic alterations in every individual analyzed, presenting out-of-range values in one or more parameters.

Significant differences were found in some parameters between the patients and controls, such as fibrinogen levels (*p* < 0.001). Interestingly, the circulating amount of fibrinogen, the G455A polymorphism in the β-chain of fibrinogen, and its interaction with tobacco smoke have been described as risk factors for LCPD [32]. This is an example of how the relationship between environmental, genetic, and metabolic factors may be related to the development of LCPD.

Because elevated levels of FIX have been established as risk factors for lower limb venous thrombosis, and given that we found higher FIX activity in the patient group (*p* < 0.001), it is presumable that FIX could play a role in the development of LCPD [33,34].

Bone homeostasis is maintained by bone-resorbing osteoclasts and bone-forming osteoblasts. The importance of maintaining bone homeostasis is underlined by diseases where imbalances in bone formation and degradation occur. In many cases, the disruption of bone homeostasis may be caused due to an imbalanced immune system. Examples of inflammatory diseases accompanied by extensive systemic and local bone degradation include rheumatoid arthritis and psoriatic arthritis. Proinflammatory interleukins have been linked to the development of osteonecrosis; therefore, it is assumed that these interleukins are related to the appearance or development of LCPD. IL23 is a proinflammatory cytokine that is recognized after the binding of IL-23R and IL-12R [17]. IL-23 is required for the maintenance, stability, and pathogenicity of Th17 cells, which are well-known key effectors of inflammation and tissue damage in several autoimmune diseases. For this reason, the IL-23R signal translation pathway has been extensively studied for its relationship with proinflammatory diseases and different pathologies that cause bone deterioration. In addition, the signaling pathway that occurs after the activity of IL-23R has multiple effects on osteoblast and osteoclast differentiation since it can inhibit or stimulate both processes [35].

Studies have also demonstrated that the absence of IL-23 also affects the physiology of bones. Regarding reduced trabecular bone mineral density, Razawy et al. showed that 7-week-old IL-23R−/− mice have a bone mass similar to age-matched littermate control mice. In contrast, 12-week-old IL-23R−/− mice have significantly lower trabecular and cortical bone mass, shorter femurs, and more fragile bones [35]. Similarly, Adamopoulos et al. proposed that IL-23 might have a role in bone remodeling [36,37]. Our results suggest a relationship between inflammation, alterations in the IL-23R, and the etiology of LCPD [38].

Homocysteine disturbances have emerged as risk factors for multiple pathological conditions, such as osteoporosis, venous thrombosis, osteonecrosis, and LCPD. In contrast, elevated Hcy levels have been associated with increased oxidative stress in bone microenvironments, which could lead to increased osteoclast differentiation and activity. Additionally, oxidative stress decreases the viability of nitric oxide through the production of superoxide anions, which would result in reduced bone blood flow and could possibly affect angiogenesis. It has been shown that osteoblast activity is affected by the concentration of Hcy, and the concentration of Hcy itself can be altered by factors such as diet and lifestyle.

The single-nucleotide polymorphism of this gene reduces the ability of the MTHFR enzyme to catalyze the conversion of 5,10-methylenetetrahydrofolate to 5-methyltetrahydrofolate and leads to a rise in plasma Hcy levels in homozygous mutated subjects, while heterozygous mutated subjects have mildly raised Hcy levels compared to normal, non-mutated controls [39,40,41,42]. Homocysteinemia has important effects on bone density and its relationship with collagen, since it interrupts the crosslinking of collagen molecules [42].

In addition, all of the members of the families with LCPD presented a mutated polymorphism in a homozygous or heterozygous manner for the *MTFHR* (rs1801133), *IL-23R* (rs1569922), and *OPG* (RS2073618) polymorphisms. Isolated cases had other genetic variants.

In conclusion, to improve our understanding of coagulation, a new convergent model of coagulation is proposed that integrates inflammation and innate immune activation as a unified response toward vascular injury. In this study, it was observed that some family-specific environmental factors, along with hemostatic and inflammatory disorders, may be involved in the development of LCPD. Our findings agree with previous studies, in which the same factors were related to the onset and development of LCPD.

In addition, since we observed familial cases and cases with genetic variants, it is very likely that inherited genetic factors have an important contribution to the onset and development of LCPD. The most significant limitation of this study is the small number of family cases described. Because LCPD is a rare disease, the prevalence of LCPD is unknown in Mexico, and many cases are underdiagnosed and undertreated. However, this is the first descriptive report of family cases in Mexico. Although we cannot establish a specific type of inheritance with this sample, we intend to examine whether different polymorphisms are related to LCPD, such as MTHFR or IL-23R. This study highlights the multifactorial nature of this pathology, involving various environmental, genetic, inflammatory, and prothrombotic factors in three families that included seven patients diagnosed with LCPD.

## 4. Methods and Materials

### 4.1. Patients

In this study, both newly diagnosed and recurrent patients of all ages were recruited and diagnosed in the Department of Pediatric Orthopedics (DPO) based on clinical criteria and radiological findings. The control group included individuals who attended the DPO and showed clinical and radiological evidence indicating the absence of femoral and hip abnormalities, with no history of thrombophilia or other conditions. Patients and controls were matched by age, sex, weight, and body mass index (BMI). All participants agreed to take part in this study, had no thrombotic pathology, and were not receiving pharmacological treatment. Both groups were recruited at the National Institute of Rehabilitation “Luis Guillermo Ibarra Ibarra” (INR-LGII) between August 2016 and April 2024. Specifically, this work was conducted in three Mexican families and included seven patients with a clinical diagnosis of LCPD and fourteen controls.

### 4.2. Controls

In parallel to this work, we studied a group of Mexican children and adolescents (controls) with plasma from 200 participants aged 0–18 with an average age of 10.7 years and a total of 112 males and 88 females. There were differences between the reagent manufacturer’s established Reference Values of Commercial Kits (RVCK) set for adults and the RVPC in this studio.

Figure 2 shows the pedigrees of the three families included in this study. Three families were included, comprising a total of 43 individuals, of whom 10 were diagnosed with LCPD (9 males and 1 female). Two patients were excluded; therefore, the final study population consisted of seven patients with LCPD. Each patient is identified by the letter P followed by a consecutive number (from P001 to P007).

According to the family’s medical history, four children were included—P002, P004, P006, and P007—with an average age of 11 ± 6.3 years and 77.9 ± 89.3 cm in height. Three male adults (the parents) were also included—P001, P003, and P005—with an average age of 43.6 ± 5.1 years and 165 ± 3 cm in height.

The figure also includes relevant clinical data. For example, there were two bilateral cases (P001 and P004), and several patients had flat feet (P003, P004, and P005) and/or practiced high-impact sports such as taekwondo (P001). Interestingly, Family 2 (Figure 2B) also had close relatives affected by osteoarthritis.

### 4.3. Biochemical Tests

A blood sample was taken from each participant and collected in a tube containing EDTA K2 and another with 3.8% sodium citrate. All hemolyzed or lipemic samples were discarded. Total blood count was performed in a Coulter LH 780 hematology (Beckman Coulter, Inc., Brea, CA, USA) automated analyzer. Citrated plasma was separated, and the samples were analyzed using commercial kits (HemosIL™ Werfen Mexico City, Mexico) in a coagulation analyzer IL ACL Elite/Pro (Instrumentation Laboratory, Bedford, MA, USA) for each determination: TP—HemosiL^TM^ PT RecombiPlasTin 2G 0020002950. The International Normalized Ratio (INR) was calculated automatically from the PT values. TTPa—HemosiL^TM^ APTT-SP (liquid) 0020006300. Factor I—HemosiL^TM^ 0008469810. Factor II—HemosiL^TM^ 0008466050. Factor V—HemosiL^TM^ 0020011500. Factor VII—HemosiL^TM^ 0020011700. Factor VIII—HemosiL^TM^ 0020011800. Factor IX—HemosiL^TM^ 0020011900. Factor X—HemosiL^TM^ 0020010000. Factor XI—HemosiLTM 0020011300. Factor XII—HemosiL^TM^ 0020201200. Antigenic von Willebrand factor—HemosiL^TM^ 0020002300. Protein C—HemosiL^TM^ 0020300500. Antithrombin (AT) liquid—HemosiL^TM^ 0020002500 and Homocysteine 0020007800 (Hcy).

Since most of the participants in this study were children, we established the proposed reference values for pediatric populations (RVPC) in accordance with the guidelines described by the International Federation of Clinical Chemistry (IFCC) and the Clinical and Laboratory Standards Institute (CLSI).

### 4.4. Molecular Tests

DNA was extracted from whole blood using a Puregene (Qiagen^TM^, Mexico City, Mexico) commercial kit, per the manufacturer’s protocol. Polymorphisms of the *MTHFR* C677T (rs1801133), *CΒS* T833C (rs115742905), *COL1A1* (rs1107946 and rs2412298), *COL2A1* (rs121912891 and rs387106558), *PT G20210A* (rs1799963), *FVL* (rs6025), *FVIII* (rs5987061), *FIX* (rs6048), *PAI-1* (rs1799889), *eNOS* (rs17899983 and rs2070744), *IL-23R* (rs1569922, rs154655686, and rs7539625), *TNF-α* (rs180062), *RANNK* (rs3018362), *RANNK-L* (rs12585014), *OPG* (rs2073618), and *IL-6* (rs1800795 and 1800796) genes were genotyped using real-time PCR with *Taqman*® probes labeled with FAM or VIC (Applied Biosystems, Foster City, CA, USA) in a Real-Time Step One PCR System (Applied Biosystems). All Taqman® probes were predesigned assays, and the primer sequences were not available from the provider.

The real-time PCR reaction mixture was performed in a volume total of 25 μL PCR containing 1X Taqman® PCR master mix, with the probe at 100 nm and with 900nm of each primer and 25 ng of genomic DNA. Cycling conditions were as follows: denaturation at 95 °C for 10 min, followed by 45 cycles at 92 °C for 15 s and then 60 °C for 1 min [43].

### 4.5. Statistical Analysis

A database in Rstudio program version 4.3 for Windows was designed for the data obtained. A comparative analysis (Mann–Whitney U test) was performed to determine any significant differences between the groups (patients and controls), *p* < 0.05.

## Figures and Tables

**Table 1:** Biochemical hemostatic markers in patients and controls.

	Participants																						
Test	P01 †^	C1	C2	P02	C3	C4	P03 †	C5	C6	P04 ^	C7	C8	P05 †	C9	C10	P06	C11	C12	P07	C13	C14	Reference Values	
																						RVPC	RVCK
Hb g/dL	**18.3**	18.0	16.2	**18.6**	15.2	15.4	**18.2**	15.3	16.3	**19.1**	16.3	16.1	**18.1**	15.8	16.7	**17.9**	14.8	14.7	**17.6**	**13.3**	13.5	13.5–17.5	12–18
FI mg/dL	414.0	275.0	267.0	**405.0**	355.0	249.0	453.0	254.0	328.0	**545.0**	219.0	250.0	395.0	299.0	221.0	**452.0**	385.0	289.0	**496.0**	**405.0**	304.0	249–360	80–700
FII %	138.0	93.7	98.8	130.0	112.0	104.0	100.0	118.0	124.0	**94.0**	**92.0**	127.0	118.0	**155.0**	112.0	130.0	107.0	**96.2**	118.0	103.0	**93.7**	98–136	50–150
FV %	**208.0**	95.2	137.0	125.0	130.0	**146.0**	146.0	150.0	115.0	134.0	**41.9**	**49.9**	146.0	55.4	95.2	117.0	**173.0**	**142.0**	133.0	**139.0**	114.0	98–136	50–150
FVII %	**173.0**	129.0	121.0	107.0	**149.0**	**141.0**	126.0	82.3	125.0	122.0	**36.8**	**184.0**	110.0	**166.0**	125.0	90.0	88.2	**134.0**	92.0	**145.0**	112.0	87–132	50–129
FVIII %	104.0	73.8	89.6	**95.0**	**116.0**	80.1	112.0	92.2	59.6	**117.0**	79.3	83.5	110.0	**35.8**	48.6	**98.0**	113.0	120.0	**99.0**	**117.0**	**107.0**	44–82	50–150
FIX %	144.0	90.8	112.0	**130.0**	**111.0**	**115.0**	**157.0**	96.8	114.0	**147.0**	94.2	95.7	132.0	72.4	82.9	**157.0**	84.1	90.8	**125.0**	67.7	89.1	73–94	65–150
FX %	**159.0**	113.0	**151.0**	118.0	101.0	130.0	121.0	121.0	**137.0**	116.0	142.0	138.0	121.0	101.0	**62.4**	111.0	105.0	136.0	107.0	121.0	128.0	100–144	77–133
FXI %	106.0	144.0	**152.0**	109.0	84.1	**144.0**	113.0	82.8	147.0	124.0	104.0	**160.0**	122.0	134.0	91.0	103.0	90.7	**186.0**	130.0	**42.8**	109.0	65–142	65–150
FXII %	91.0	50.0	90.8	104.0	61.2	**118.0**	108.0	50.0	134.0	81.0	86.8	**195.0**	81.0	146.0	96.0	104.0	57.5	**156.0**	93.0	67.4	95.6	55–109	50–150
VWF %	82.0	66.0	**38.3**	**48.0**	90.5	**28.0**	104.0	66.0	80.0	124.0	124.0	**134.0**	72.0	80.9	137.0	**50.0**	120.0	**49.1**	**54.0**	85.8	**50.7**	69–130	66–170
PC %	110.0	101.0	117.0	102.0	109.0	91.8	85.0	121.0	99.3	87.0	124.0	118.0	**147.0**	122.0	119.0	**130.0**	92.4	90.4	**135.0**	120.0	**76.0**	83–128	70–140
AT %	108.0	123.0	**134.0**	121.0	120.0	135.0	**81.0**	119.0	116.0	122.0	134.0	103.0	**147.0**	126.0	92.0	130.0	142.0	144.0	135.0	119.0	**147.0**	108–144	83–128
Hcy µmol/L	**13.1**	11.1	10.3	7.2	8.5	9.7	**14.2**	8.1	3.1	9.7	**3.5**	4.1	6.5	6.2	7.7	9.4	5.1	6.9	7.6	6.4	7.7	4–11.2	4–11.2

P: patients. C: controls (gray shading). Hb: hemoglobin. F: coagulation factor. VWF: Von Willebrand factor. PC: protein C. AT: antithrombin. Hcy: homocysteine. RVPC: reference values proposed for children, bold values are all those which were out of range. RVCK: reference values of commercial kits. †: adult. ^: bilateral disease. g/dL = grams per deciliter. mg/dL = milligrams per deciliter. μmol/L = micromoles per liter. % = percentage of activity. Mann-Whitney U with the Rstudio program version 4.3, *p* < 0.001.

**Table 2:** Molecular markers in LCPD patients.

Gene	rs	Wild Type	Mutant Type	P01 †^	P02	P03 †	P04 ^	P05 †	P06	P07
*MTHFR*	1801133	C	T	T/**T**	C/**T**	C/**T**	C/**T**	C/**T**	T/**T**	C/**T**
*CBS*	115742905	T	C	T/T	T/T	T/T	T/T	T/T	T/T	T/T
*COL1A1*	1107946	G	T	G/**T**	T/**T**	G/G	G/G	G/G	G/G	G/G
	2412298	C	T	C/**T**	C/C	C/**T**	C/C	C/C	C/**T**	C/**T**
	1800012	C	C	C/C	C/C	C/C	C/C	C/C	C/C	C/C
	2412293	G	A	G/G	G/G	G/**A**	G/G	G/**A**	G/**A**	G/**A**
*COL2A1*	121912891	G	A	G/**A**	G/G	G/G	G/G	G/G	G/G	G/G
	387106558	G	A	G/**A**	G/G	G/G	G/G	G/G	G/G	G/G
*PT*	1799963	G	A	G/G	G/G	G/G	G/G	G/G	G/G	G/G
*FVL*	6025	C	T	C/**T**	C/C	C/C	C/C	C/C	C/C	C/C
*FVIII*	5987061	C	T	C/**T**	C/C	C/C	C/C	C/C	C/C	C/C
*FIX*	6048	A	G	A/**G**	A/A	A/A	A/A	A/A	A/**G**	A/**G**
*PAI-1*	1799889	A	G	A/**G**	A/A	A/A	A/A	A/A	A/A	A/A
*eNOS*	17899983	G	T	G/**T**	G/G	G/G	G/G	G/G	G/G	G/G
	2070744	T	C	T/**C**	T/T	T/T	T/T	T/**C**	T/T	T/T
*IL-23R*	1569922	C	T	C/**T**	C/**T**	C/**T**	C/**T**	C/**T**	**T**/**T**	C/**T**
	154655686	G	A	G/**A**	G/G	G/G	G/G	G/G	G/G	G/G
	7539625	G	A	G/**A**	G/G	G/**A**	G/G	**A/A**	G/G	G/G
*TNF-α*	180062	G	A	G/**A**	G/G	G/G	G/G	G/G	G/G	G/G
*RANNK*	3018362	G	A	**A**/**A**	**A**/**A**	**A**/**A**	**A**/**A**	G/G	G/**A**	G/**A**
*RANNK-L*	12585014	G	A	G/G	G/**A**	G/G	G/**A**	G/G	G/**A**	G/G
*OPG*	2073618	C	G	G/**C**	G/**C**	G/**C**	G/**C**	G/**C**	**C**/**C**	**C**/**C**
*IL-6*	1800795	G	C	G/**C**	G/G	G/G	G/G	**C**/**C**	**C**/**C**	G/**C**
	1800796	G	C	G/G	G/**C**	G/**C**	G/**C**	G/G	G/G	G/G

P: Patient. † = adult. ^ = bilateral disease. A = Adenine. T = Thymine. G = Guanine. C = Cytosine. rs: reference SNP. *MTHFR* = methylenetetrahydrofolate reductase. *PT* = prothrombin. *CBS* = cystathionine beta-synthase. *COL1A1* = type IA collagen. *COL2A1* = type IIA collagen. *FVL* = Factor V Leiden. *FVIII* = Factor VIII. *FIX* = Factor IX. *PAI-1* = Plasminogen activator inhibitor-1. *eNOS* = Endothelial nitric oxide synthase. *IL-23R* = Interleukin-23 receptor. *TNF-α* = tumor necrosis factor Alpha. *RANNK* = Receptor Activator of Nuclear Factor κ B. *RANNK-L* = Receptor Activator for Nuclear Factor κ B Ligand. *OPG* = Osteoprotegerin. *IL-6* = Interleukin-6.

## Data Availability

The original contributions presented in this study are included in the article. Further inquiries can be directed to the corresponding authors.
